# Simultaneous Onset of Pediatric Systemic Lupus Erythematosus in Twin Brothers: Case Report

**DOI:** 10.3389/fped.2022.929358

**Published:** 2022-06-16

**Authors:** Rinat K. Raupov, Evgeny N. Suspitsin, Artur I. Imelbaev, Mikhail M. Kostik

**Affiliations:** ^1^Hospital Pediatry Department, St. Petersburg State Pediatric Medical University, Saint Petersburg, Russia; ^2^H. Turner National Medical Research Center for Children's Orthopedics and Trauma Surgery, Saint Petersburg, Russia; ^3^City Hospital, Saint Petersburg, Russia; ^4^N. N. Petrov Institute of Oncology, Molecular Diagnostics, Saint Petersburg, Russia; ^5^Molecular Genetics Department, St. Petersburg State Pediatric Medical University, Saint Petersburg, Russia; ^6^Radiology Department, St. Petersburg State Pediatric Medical University, Saint Petersburg, Russia

**Keywords:** pediatric systemic lupus erythematosus, twins, antiphospholipid syndrome, IFN-signature, RNASEL

## Abstract

There are hundreds of twin adult patients with systemic lupus erythematosus (SLE), but male children with SLE are rarely affected. Two monozygotic twin brothers developed SLE at the age of 11 years during 1 month. The index brother manifested with Henoch-Shonlein purpura, accompanied by ANA positivity, and later developed critical left femoral arterial stenosis with high levels of anti-dsDNA, antiphospholipid antibodies, hypocomplementemia, and Coombs-positive hemolytic anemia. At that time his twin brother had only identical autoimmune findings and developed clinical manifestation (myositis and fasciitis) a month later. Both twins had increased IFN-score and shared a heterozygous variant in the RNASEL gene. Index patients developed scalp rash and nephritis 6 months after their parents refused the treatment which has been lasted for 1 year after disease diagnostics.

**Conclusion:**

The simultaneous onset of the pediatric SLE in the male twin is a very rare situation suspected monogenic origin of the disease. Further functional studies are required to confirm the causative role of the mutation.

## Introduction

Familial cases of SLE are known, but just a few hundred cases of SLE in twins have been described in the world. Usually, SLE affects monozygotic female twins, while the descriptions of SLE in twin males are rare (1). Several genetic variants associated with lupus have been previously described. High penetrant single gene variants leading to complement deficiency (C1Q, C1R/S, C2, C4A, and C4B) are rare and mostly presented in patients with early-onset SLE while plenty of common low penetrant variants may only increase the risk of SLE. The following pathways are predominantly involved in SLE pathogenesis: TLR/IFN-I signaling, NF-κB signaling, T-cell signaling, B-Cell signaling, T- and B-Cell signaling and interaction, self-antigen clearance, IC clearance, DNA repair (2). This diversity explains the clinical and genetic heterogeneity of SLE. Some associations between gene defects and clinical manifestations have been found (3).

## Case Description

Two 11^th^ years monozygotic twin boys developed SLE within 1 month. Family medical history is unremarkable, except cold-induced allergy of the maternal grandfather. No previous immune dysregulation diseases were observed in the twins.

### Index Twin

**Figure 1:**
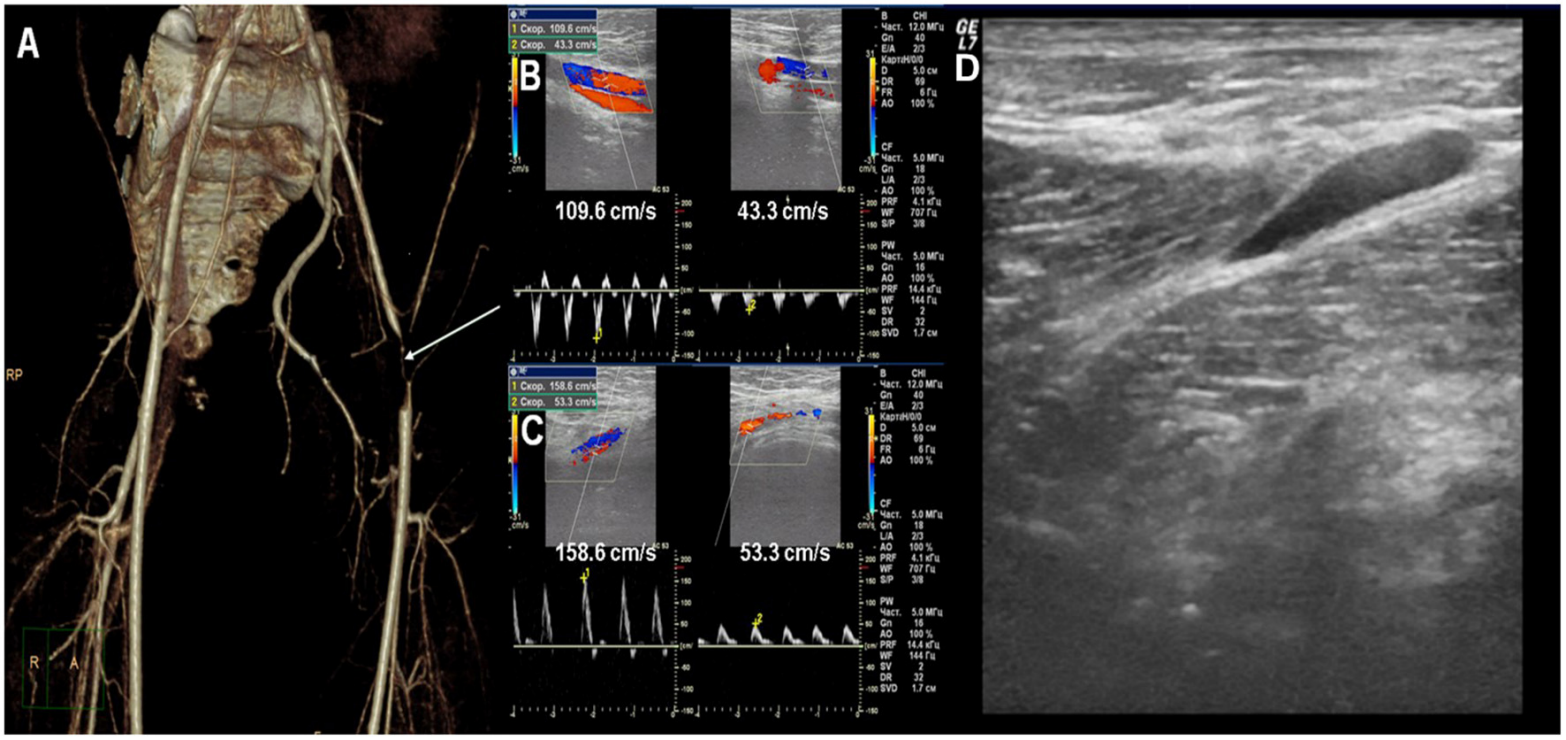
**(A)** CT-angiography: critical stenosis of the left femoral artery. **(B)** Doppler ultrasound: the difference of blood flow velocity in the iliac arteries. **(C)** Doppler ultrasound: the difference of blood flow velocity in the femoral arteries. **(D)** Ultrasound: fascia thickening of the quadriceps and sub-fascial fluid accumulation.

Twin 1 had pain in the legs after physical activity for 6 months and pain in the groin during walking for the last month before the hospital admission. Then hemorrhagic rash on the legs, the left ankle, and the wrists edema, and fever appeared. He was admitted to the local hospital and Henoch-Schonlein purpura was diagnosed. Laboratory tests showed inflammation with mild leukocytosis and neutrophilia, ESR and CRP elevation, transaminase elevation, and positive direct Coombs test. Infections were ruled out. He was treated with antibiotics and heparin. Fever and rash were resolved, while the left ankle edema persisted. Due to ANA-positivity (1:5120, NR <1:160) he was transferred to our rheumatologic clinic. Suddenly he developed intermittent claudication, pain in the left calf muscle, and hypothermia on the left foot. The discrepancy in the blood pressure between the legs was detected on physical examination. He had mild ESR elevation, coagulopathy, hypocomplementemia, positive direct Coombs test without signs of hemolysis, and very high autoimmune activity with ANA positivity, anti-dsDNA, and antiphospholipid autoantibodies (Table 1). CT-angiography detected critical stenosis of the left femoral artery (Figure 1A). Doppler-ultrasound showed the difference in the blood flow pattern in the left iliac and the femoral arteries (Figures 1B,C). He was effectively treated with oral corticosteroid (prednisolone 1 mg/kg), hydroxychloroquine, IV prostaglandin E1, and low-weight heparin followed by warfarin. The symptoms resolved, signs of stenosis and arteritis partially improved and steroid tapering was recommended.

**Table 1:** Laboratory characteristics of patients at the disease onset.

**Studied parameter**	**Twin 1**	**Twin 2**
Hemoglobin, g/dl (n.v 11.0–16.5)	11.4	11.5
Leucocytes, 10^9^/l (n.v. <4.0–10.0)	4.3	5.5
Thrombocytes, 10^9^/l (n.v.180–400)	141	182
ESR, mm/h (n.v. <20)	20	9
CRP, mg/l (n.v. <5)	1.4	3.4
Activated partial thromboplastin time, sec (n.v.25.1–36.3)	136	82.1
D-dimer, ng/ml (n.v. <250)	765	1,029
C3 complement, g/l (n.v. 0.82–1.73)	0.84	0.84
C4 complement, g/l (n.v.0.13–0.46)	0.05	0.06
Lupus anticoagulant (n.v.0.8–1.2)	3.06	2.86
Coombs test	Positive	Positive
Antinuclear antibody (n.v. <1:160)	1:5,120	1:5,120
Anti-dsDNA, IU/ml (n.v. <25)	>800	>800
Anticardiolipin antibody*, IgG, GPL-U/ml (n.v. <12)	45.8	109.5
Anticardiolipin antibody*, IgM, MPL-U/ml (n.v. <12)	99.8	>120
Anti-β2-glycoprotein I*, MPL-U/ml (n.v. <20)	>200	>200

n.v., normal value, *all antiphospholipid antibodies were positive throw 12 weeks in both patients.

### Twin 2

Twin 2 has no symptoms at the moment of Twin 1 clinical manifestation. The parents decided to examine him and identical laboratory and autoimmune changes as in Twin 1 were found (Table 1). The low dose of acetylsalicylic acid with hydroxychloroquine was started due to the presence of positive antiphospholipid antibodies for thrombotic events profilaxis. 1 month after Twin 1 clinical manifestation Twin 2 had acute severe pain in the left leg with hemorrhagic rash (petechiae) in the legs. The left lower leg circumference (mid-thigh) was +1.5 cm than the right one, painful on palpation. Laboratory tests showed the signs of inflammation (ESR 47 mm/h, CRP 20.1 mg/l), hypocomplementemia, and coagulopathy [APPT 45.1 sec (↑), D-Dimer 770 ng/ml (↑)]. Doppler ultrasound has not revealed any blood vessels pathology, except the fascia thickening of the quadriceps and fluid accumulation (Figure 1D). Corticosteroids (prednisolone 1 mg/kg) were immediately started with a resolution of clinical symptoms and laboratory parameters normalization and further tapered.

## Genetic Testing

IFN I-score was assessed by RT-PCR quantitation of 5 IFN I-regulated transcripts (IFI44L, IFI44, IFIT3, LY6E, MXA1); median relative expression of ≥ 2 was considered as a cut-off. Both twins had elevated blood IFN-I expression scores (9.9 and 10.0, normal range 0.57–1.9) in the second visit on corticosteroid treatment. Clinical exome sequencing of the twins revealed a shared heterozygous variant, RNASEL c.1880A>G (p.K627R), rs149964724. This substitution has a MAF (minor allele frequency) of 0.000378 (Genome Aggregation Database; gnomAD); it has not been reported in ClinVar. According to the ACMG (American College of Medical Genetics and Genomics) criteria the variant may be classified as Likely benign (BP1, BP4). The variant was inherited from an asymptotic mother.

## Further Follow-Up

**Figure 2:**
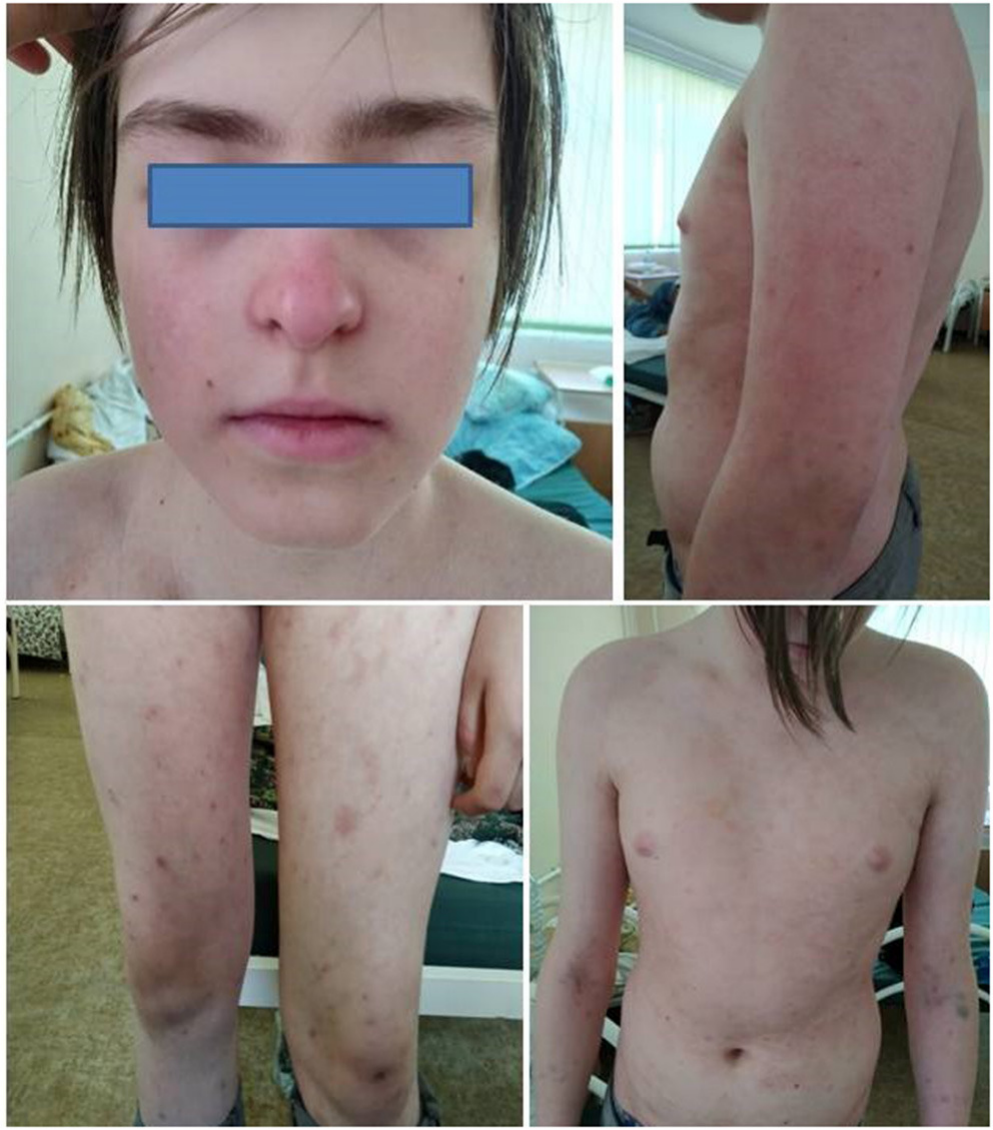
The rash in Twin 1 during the flare (Month 30 from the onset).

Corticosteroids have been tapered up to 0.2 mg/kg during the following 6 months without flare in both twins. Both patients also had migraine headaches, the levels of anti-dsDNA and antiphospholipid antibodies still had increased in several scheduled testing in the following 18 months. Brain MRI was normal. Rituximab was recommended, but the parents refused it as well as any other treatment. In the 24 months from the onset, the parents refused the following treatment in both twins. Twin 1 developed intensive skin and scalp rash, thrombocytopenia (PLT 72 x 10^9^/l), and lupus nephritis (proteinuria 1,1 g/24 h) 6 months after the treatment ceased (Figure 2). Despite clinical and laboratory deterioration, his IFN-I score was lower (5.7) than in the disease onset (9.9). Oral corticosteroids 1 mg\kg were initiated, but parents have been against the kidney biopsy and any additional treatment (rituximab or synthetic DMARDs). Twin 2 hasn't developed the symptoms of SLE for the following 3 years.

## Discussion

We described a rare case of the simultaneous onset of pediatric SLE in male monozygotic twins: Twin 1 has developed a typical SLE with antiphospholipid syndrome and his brother Twin 2 has not realized yet full-blown SLE possibly due to early diagnostics and treatment intervention. Local arterial involvement is a rare phenomenon in lupus. It was difficult to distinguish local femoral arteritis from arterial thrombosis without distal ischemic lesions in Twin 1. Twin 2 manifested with similar local thigh changes, but it is unclear whether the early treatment of Twin 2 prevented vascular lesions or not. According to ACR/EULAR 2019 Twin1 meets SLE criteria (totally 19 points). Twin 2 scored 11 points by only immunologic features without pronounced clinical manifestation.

The first investigations proposing the genetic background of SLE were performed for twins in the second part of the XX century (4). The presence of SLE and autoantibodies production in concordant and discordant twins were analyzed. The literature review showed 60/151 concordant SLE cases in monozygotic twins while only 4/96 dizygotic (1). Population-based Danish study demonstrated concordance rates of 25% in monozygotic and 7.7% in dizygotic SLE twins. Only 2/22 (9.1%) were male twins with SLE. The majority of twins (19/22) developed clinical manifestations of SLE within the same year (5). Serologic studies were performed on 7 pairs of identical twins: three were concordant and four were discordant for SLE. Concordant twins had similar autoantibodies and anti-RNA profiles, whereas affected twins had higher titers than unaffected discordant pairs. The profile of anti-RNA proteins (e.g., Ro/SS-A, La/SS-B, U1 RNP, and Sm) was identical in 3/4 of discordant twins (6). First-degree relatives of SLE patients had higher ANA autoantibodies levels than healthy controls (7). Autoantibodies against a panel of 21 autoantigens (excluding anti-dsDNA and antiphospholipid autoantibodies) were analyzed in discordant twin patients with SLE (*n* = 9) and idiopathic inflammatory myositis (*n* = 22). Only 3/31 (10%) unaffected twins were seropositive against single autoantigens not observed in twins with the disease (8). The results of these studies indicate SLE heterogeneity and the role of genetic and epigenetic factors.

Our patients have a variant in the gene which might play role in the interferon type I signaling. The hyperactivation of the interferon I signaling pathway plays a major part in lupus pathogenesis and several medications affecting IFNI hyperproduction had been recently tested (tofacitinib, baricitinib, anifrolumab, sifalimumab, IFN-αkinoid, BIIB059) (9). Type I IFNs stimulate both the innate and adaptive immune systems, which contribute to loss of tolerance and a sustained autoimmune disease process (10). There are many variants in genes that encode components of the IFNI pathway associated with SLE. Some single gene variants (SAMHD1, RNASEH2ABC, ADAR1, IFIH1, ISG15, ACP5, TMEM173) can cause monogenic lupus (11). They are characterized by early SLE onset and severe disease manifestation. Monogenic lupus has similar features to type I interferonopathies and some interferonopathies, such as CANDLE, SPENCD, SAVI, Aicardi-Goutières syndrome has lupus as a part of the disease (12).

In the recent paper, the whole-exome sequencing (WES) was performed in 52/281 pediatric SLE cases who fulfilled one of the following criteria: early onset of the disease (<5 years), family history of autoimmune disease and complicated conditions, and causative mutations in 5 genes (SLC7A7, NRAS, TNFAIP3, PIK3CD, and IDS) were identified in 12 patients (23.1%) (13).

Seven children with SLE who had a disease onset ≤ 5 years and a family history consistent with an autosomal recessive inheritance were enrolled in the next study. WES revealed two novel and three previously reported homozygous variants in genes coding early complement proteins (C1QA, C1QC, and C1S), and one patient had a DNASE1L3 variant, which might activate interferon type I signaling (14).

Tirosh, et colleagues performed WES in 6/15 newly diagnosed childhood-onset SLE with severe (life-threatening or organ-threatening presentation), atypical presentation (out of the typical clinical classification criteria for SLE), close-married parents, or additional comorbidities (i.e., immunodeficiency). Four unrelated severe cases of childhood-onset SLE were secondary to mutations in five different genes, the last three of them are working in interferon type I signaling: C1QC, SLC7A7, MAN2B1, PTEN, and STAT1 (15). Ultra-rare (≤0.1%) missense and non-sense variants in 22 genes, known to cause monogenic forms of SLE were identified in 71 SLE patients and their healthy parents with WES. Only one homozygous mutation in C1QC and seven heterozygous variants in five genes (C1S, DNASE1L3, DNASE1, IFIH1, and RNASEH2A), associated with monogenic SLE were identified (16). All genes, except for C1S are involved in interferon type I signaling.

In a recent Chinese study, WGS were analyzed in three families where 7 of 16 members had SLE. There was one discordant for SLE female pair. The pathogenic risk of rare missense variants in WNT 16 and ERVW-1 genes was identified in five and two patients, respectively. Whereas, none of the healthy family members has mutations (17). Genome-wide DNA methylation changes in sorted CD4 + T-cells, monocytes, granulocytes, and B-cells were analyzed in 15 SLE-affected twin pairs. All cell types had hypomethylation in interferon-regulated genes, such as IFI44L, PARP9, and IFITM1. Notably, hypomethylation was more pronounced in patients who had disease flare within the past 2 years. In contrast to the other cell types, differentially methylated CpGs in B -cells were predominantly hypermethylated, were the most important upstream regulators included TNF and EP300 (18). The case of monozygotic twins discordant for SLE was described. A total of 8 putative discordant variants in the DNA were selected out for validation by Sanger sequencing, but all of them were ultimately non-diagnostic (19).

It's known that IFN type I hyperactivation leads to endothelial dysfunction and activates the coagulation cascade, which may explain arterial involvement (10). A recent study demonstrated that type I IFN score was increased in all the patients with primary antiphospholipid syndrome and antiphospholipid syndrome in SLE (20). RNASEL gene encodes a ribonuclease participating in IFN induction. While RNA/DNA-modulating enzymes are known to play a role in lupus pathogenesis (2), it is unclear whether the RNASE variant, which has been found in our twins is associated with our patients' phenotype or not.

## Conclusion

SLE in male twins is a rare and unique phenomenon. Genetic factors probably play a crucial role in such cases. There're only a few studies where WGS/WES were performed in twins with SLE. It is necessary to study the spectrum of genetic variants associated with SLE.

## Data Availability Statement

The original contributions presented in the study are included in the article/supplementary material, further inquiries can be directed to the corresponding author/s.

## Ethics Statement

Ethical review and approval was not required for the study on human participants in accordance with the local legislation and institutional requirements. Written informed consent to participate in this study was provided by the participants' legal guardian/next of kin.

## Author Contributions

MK and RR had full access to all of the data in the study and takes responsibility for the integrity of the data and the accuracy of the data analysis. All authors were involved in drafting the article or revising it critically for important intellectual content, and all authors approved the final version to be published.

## Funding

This work was supported by the RSF Grant No: 20-45-01005.

## Conflict of Interest

The authors declare that the research was conducted in the absence of any commercial or financial relationships that could be construed as a potential conflict of interest.

## Publisher's Note

All claims expressed in this article are solely those of the authors and do not necessarily represent those of their affiliated organizations, or those of the publisher, the editors and the reviewers. Any product that may be evaluated in this article, or claim that may be made by its manufacturer, is not guaranteed or endorsed by the publisher.

## References

[1] Block SR. A brief history of twins. Lupus. (2006) 115:61-64. 10.1191/0961203306lu2263ed 16539274

[2] Deng Y Tsao BP. Genetics of Human SLE. Ninth Edition: Elsevier Inc.

[3] Lennard Richard ML Tsao BP. Genes and Genetics in Human SLE. Second Edition: Elsevier Inc.

[4] Block SR Lockshin MD Winfield JB Weksler ME Imamura M Winchester RJ. Immunologic observations on nine sets of twins either concordant or discordant for SLE. Arthritis Rheum. (1976) 119:545-554. 10.1002/art.1780190306 132937

[5] Ulff-Møller CJ Svendsen AJ Viemose LN Jacobsen S. Concordance of autoimmune disease in a nationwide Danish systemic lupus erythematosus twin cohort. Semin Arthritis Rheum. (2018) 47:538-544. 10.1016/j.semarthrit.2017.06.007 28755788

[6] Reichlin M Harley JB Lockshin MD. Serologic studies of monozygotic twins with systemic lupus erythematosus. Arthritis Rheumatol. (1992) 4:457–464.. 10.1002/art.1780350416 1567495

[7] Navarra SV Ishimori ML Uy EA Hamijoyo L Sama J James JA. Studies of Filipino patients with systemic lupus erythematosus: autoantibody profile of first-degree relatives. Lupus. (2011) 20:537–543. 10.1177/0961203310385164 21183559

[8] Gan L O'Hanlon TP Gordon AS Rider LG Miller FW Burbelo PD. Twins discordant for myositis and systemic lupus erythematosus show markedly enriched autoantibodies in the affected twin supporting environmental influences in pathogenesis. BMC Musculoskelet Disord. (2014) 15:67. 10.1186/1471-2474-15-67 24602337 PMC3973849

[9] Klavdianou K Lazarini A Fanouriakis A. Targeted biologic therapy for systemic lupus erythematosus: emerging pathways and drug pipeline. Bio Drugs. (2020) 34:133. 10.1007/s40259-020-00405-2 32002918

[10] Rönnblom L Leonard D. Interferon pathway in SLE: one key to unlocking the mystery of the disease. Lupus Sci Med. (2019) 6:1–11. 10.1136/lupus-2018-000270 31497305 PMC6703304

[11] Costa-Reis P Sullivan KE. Monogenic lupus: it's all new! *Current Opinion Immunol*. (2017) 49:87–95. 10.1016/j.coi.2017.10.008 29100097

[12] Melki I Frémond ML. Type I Interferonopathies: from a novel concept to targeted therapeutics. Current Rheumatol Rep. (2022) 22:1–4. 10.1007/s11926-020-00909-4 32548765

[13] Li G Liu H Li Y Zhang T Yao W Guan W. Genetic heterogeneity in Chinese children with systemic lupus erythematosus. Clin Exp Rheumatol. (2021) 39:214–222. 33337996 10.55563/clinexprheumatol/zte897

[14] Batu ED Koşukcu C Taşkiran E Sahin S Akman S Sözeri B. Whole exome sequencing in early-onset systemic lupus erythematosus. J Rheumatol. (2018) 45:1671–671. 10.3899/jrheum.171358 30008451

[15] Tirosh I Spielman S Barel O Ram R Stauber T Paret G. Whole exome sequencing in childhood-onset lupus frequently detects single gene etiologies. Pediatr Rheumatol Online J. (2019) 17:52. 10.1186/s12969-019-0349-y 31362757 PMC6668194

[16] Almlöf JC Nystedt S Leonard D Eloranta ML Grosso G Sjöwall C. Whole-genome sequencing identifies complex contributions to genetic risk by variants in genes causing monogenic systemic lupus erythematosus. Human Gen. (2019) 138:141–50. 10.1007/s00439-018-01966-7 30707351 PMC6373277

[17] Chen J Zhang P Chen H Wang X He X Zhong J. Whole-genome sequencing identifies rare missense variants of WNT16 and ERVW-1 causing the systemic lupus erythematosus [published online ahead of print, 2022 Mar 10]. Genomics. (2022) 114:110332. 10.1016/j.ygeno.2022.110332 35283196

[18] Ulff-Møller CJ Asmar F Liu Y Svendsen AJ Busato F Grønbæk K. Twin DNA methylation profiling reveals flare-dependent interferon signature and b cell promoter hypermethylation in systemic lupus erythematosus. Arthritis Rheumatol. (2018) 70:878. 10.1002/art.40422 29361205

[19] Chen F Li Z Li R Li Y. Whole-genome sequencing of a monozygotic twin discordant for systemic lupus erythematosus. Mol Med Rep. (2018) 17:8391–391. 10.3892/mmr.2018.8912 29693174

[20] Palli E Kravvariti E Tektonidou MG. Type I interferon signature in primary antiphospholipid syndrome: clinical and laboratory associations. Front Immunol. (2019) 10:1–7. 10.3389/fimmu.2019.00487 30930907 PMC6428719

